# Supplementary material for: The Multifaceted Role of EXOC6A in Ciliogenesis
Source: bioRxiv. 2025 Jul 25:2025.07.25.666797. Preprint. [Version 1] doi: 10.1101/2025.07.25.666797 (PMC12330657; doi:10.1101/2025.07.25.666797)

Supplementary Figure 1

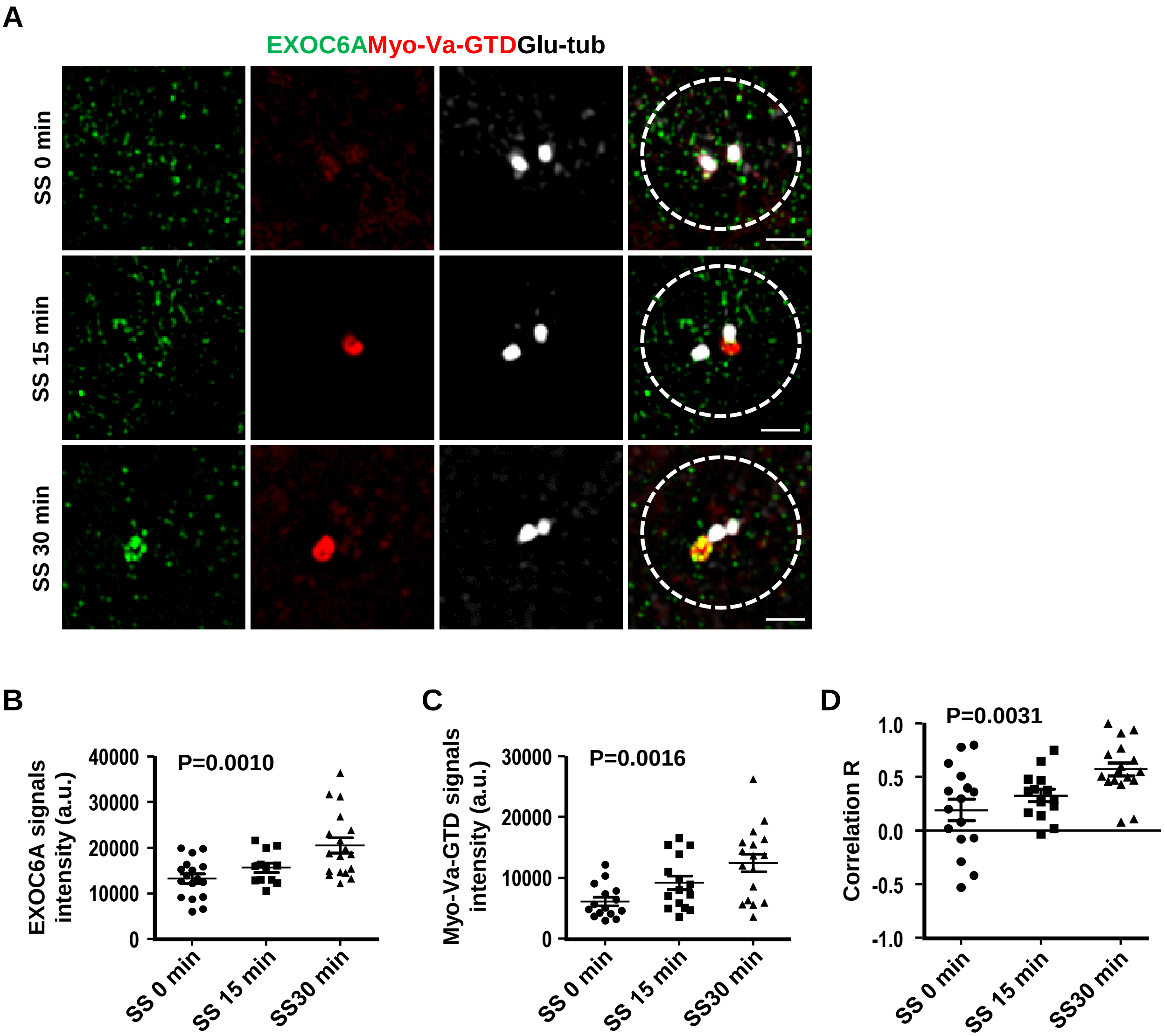

Supplementary Figure 2

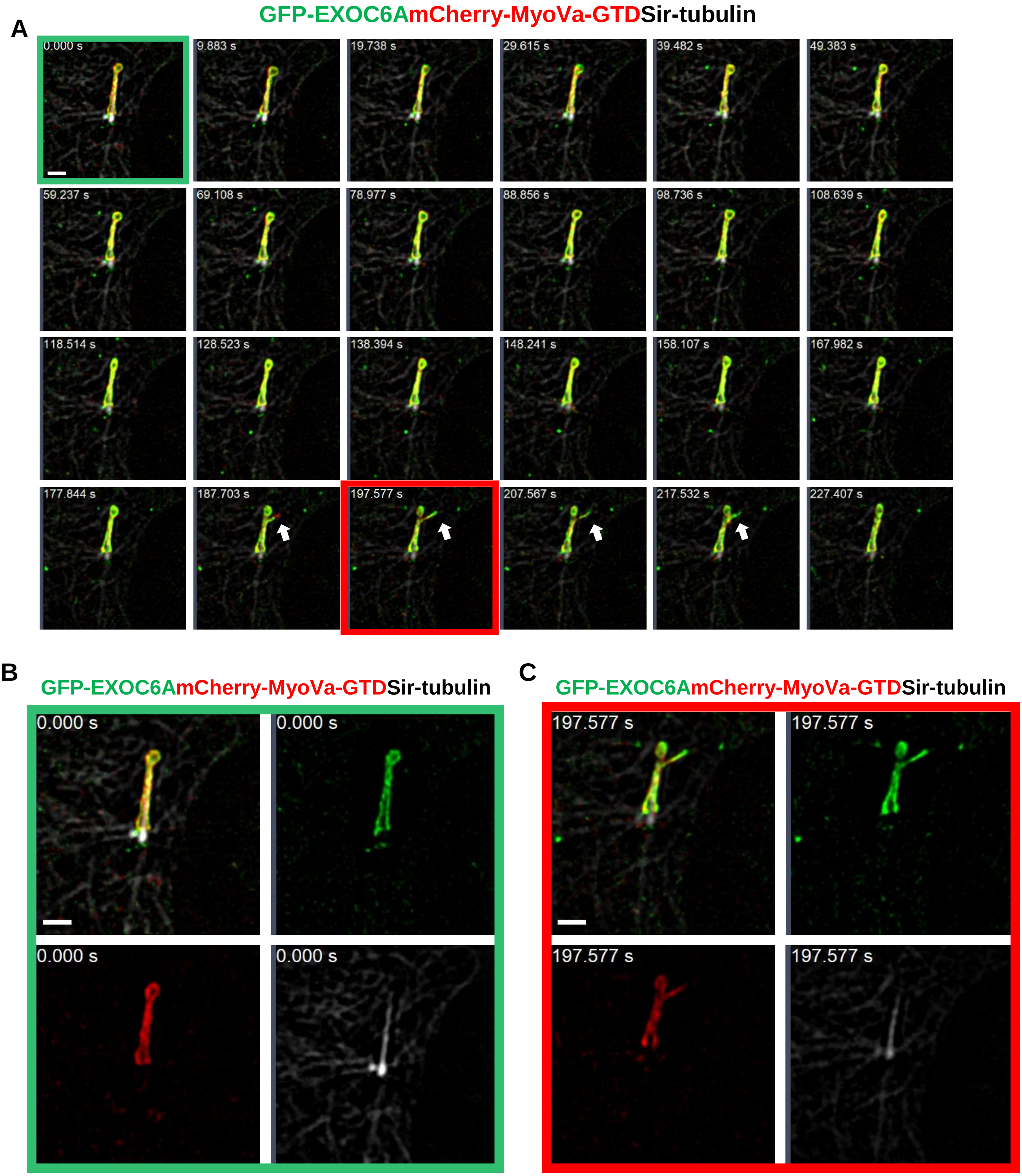

Supplementary Figure 3

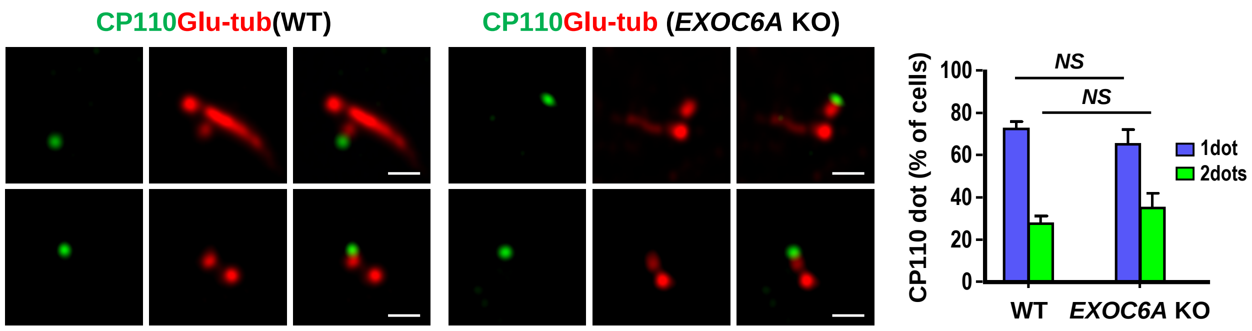

Supplementary Figure 4

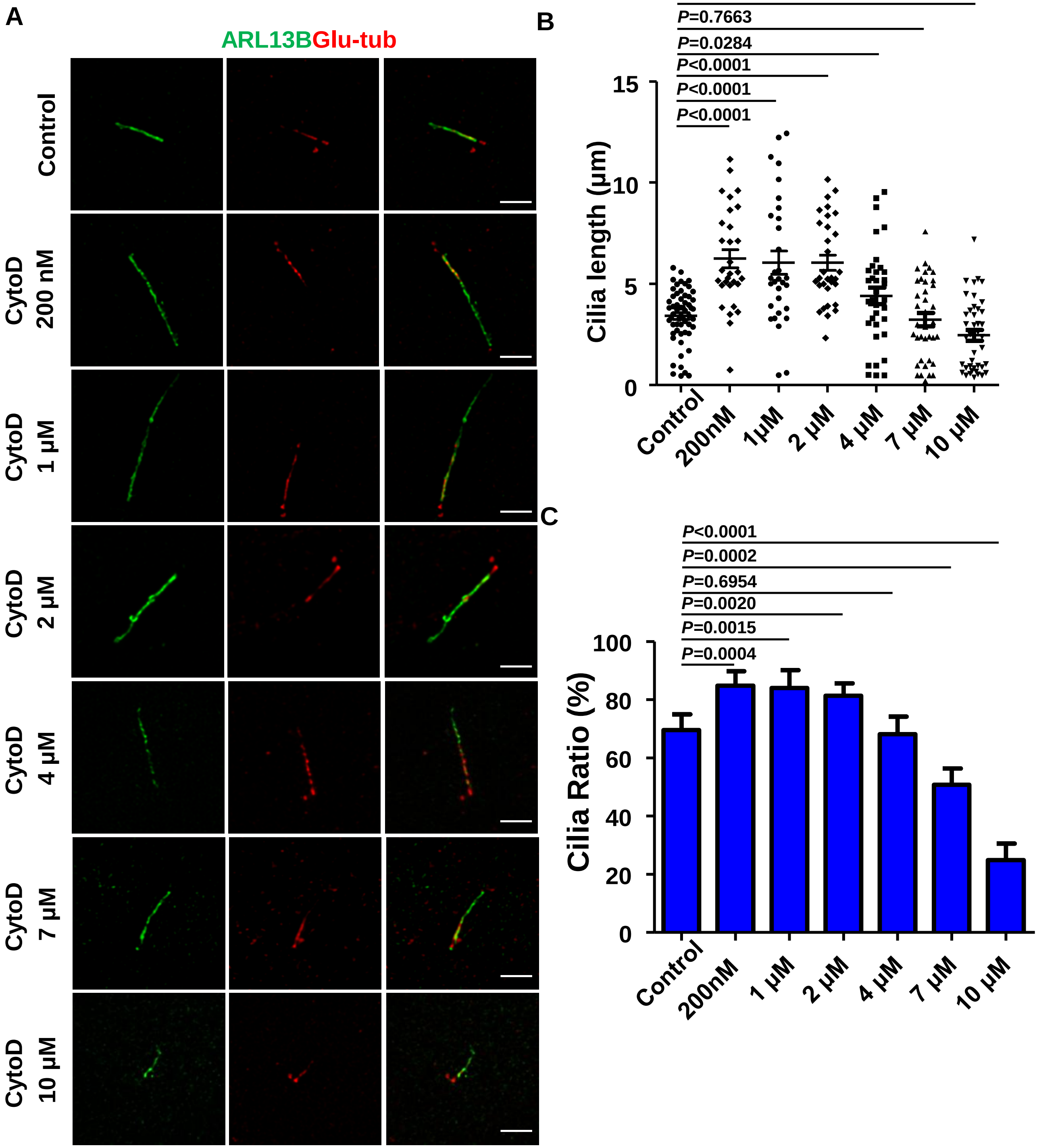

Supplementary Figure 5

A

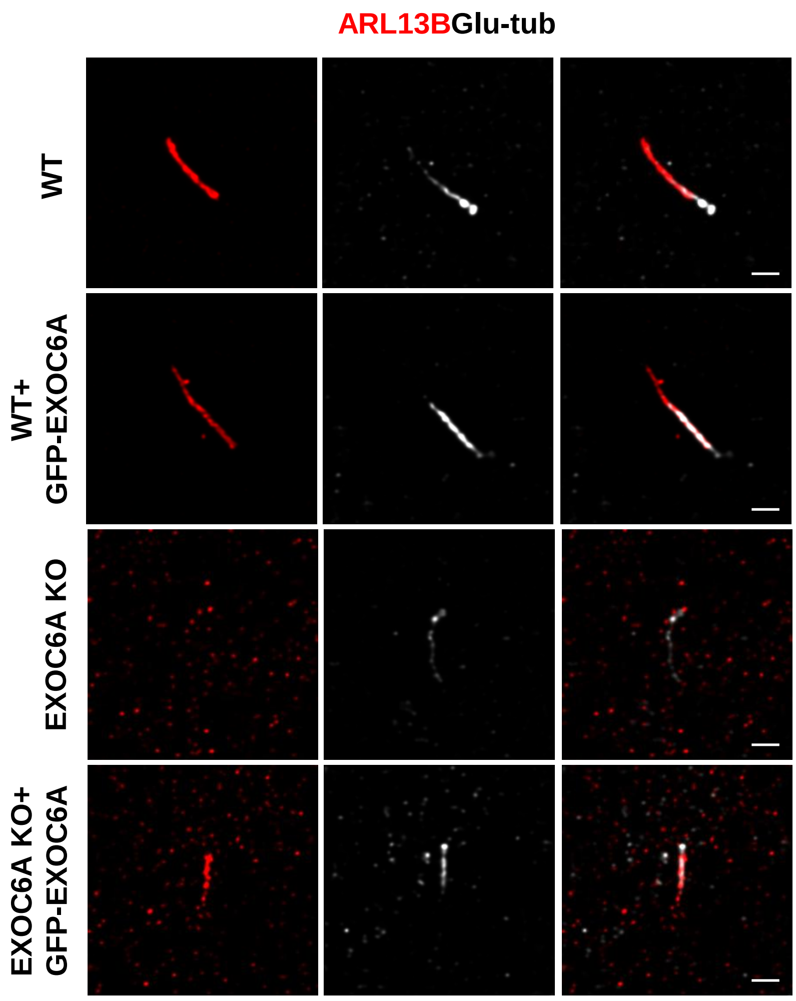

B

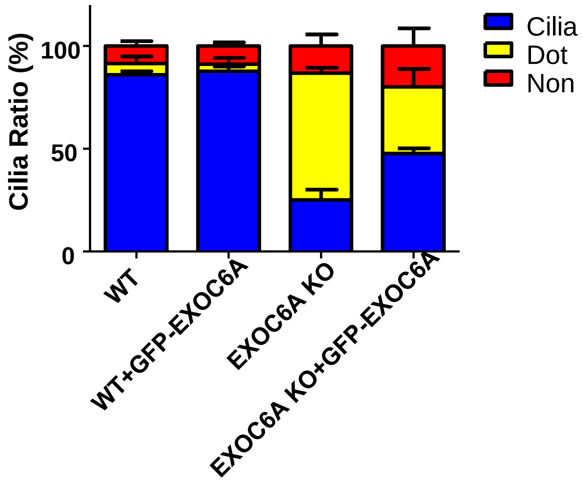

Source Data

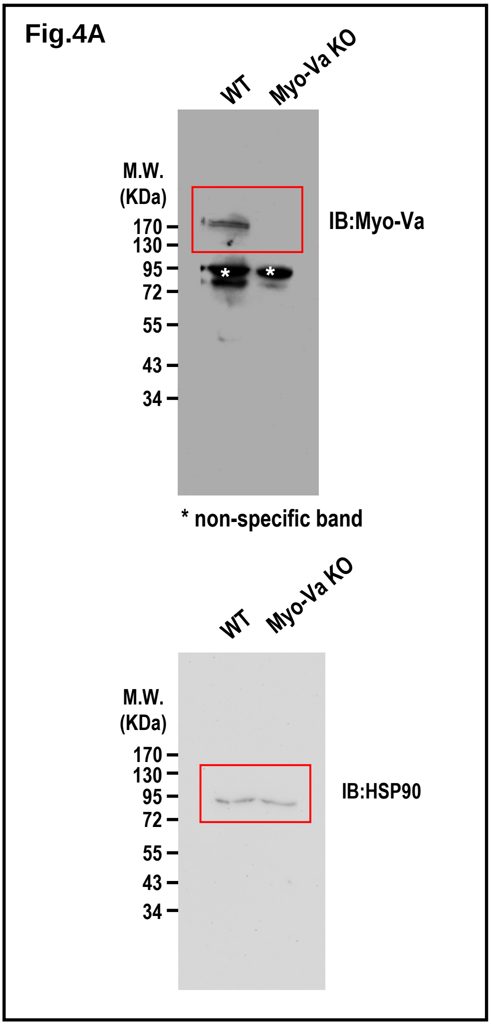

Fig.4A
WT
Myo-Va KO
M.W.
(KDa)
170
130
95
72
55
43
34
IB:Myo-Va
* non-specific band
WT
Myo-Va KO
M.W.
(KDa)
170
130
95
72
55
43
34
IB:HSP90

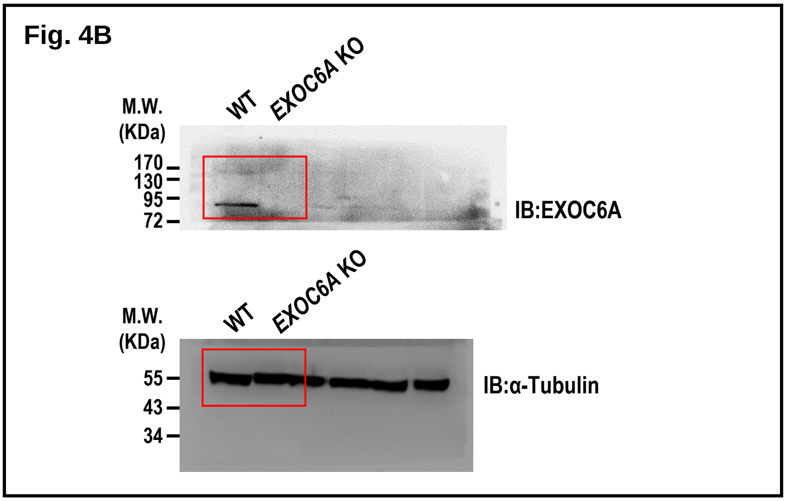

Fig. 4B
M.W.
(KDa)
WT
EXOC6A KO
170
130
95
72
IB:EXOC6A
M.W.
(KDa)
WT
EXOC6A KO
55
43
34
IB:α-Tubulin

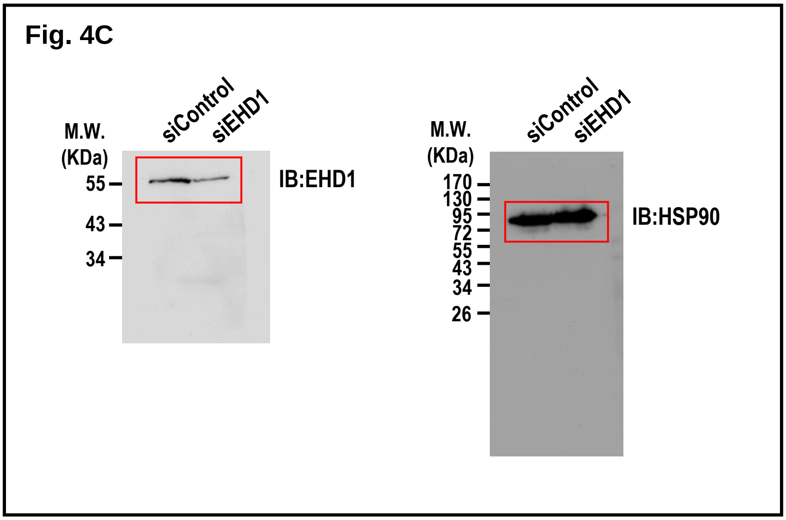

Fig. 4C
M.W.
(KDa)
siControl
siEHD1
55
43
34
IB:EHD1
M.W.
(KDa)
siControl
siEHD1
170
130
95
72
55
43
34
26
IB:HSP90

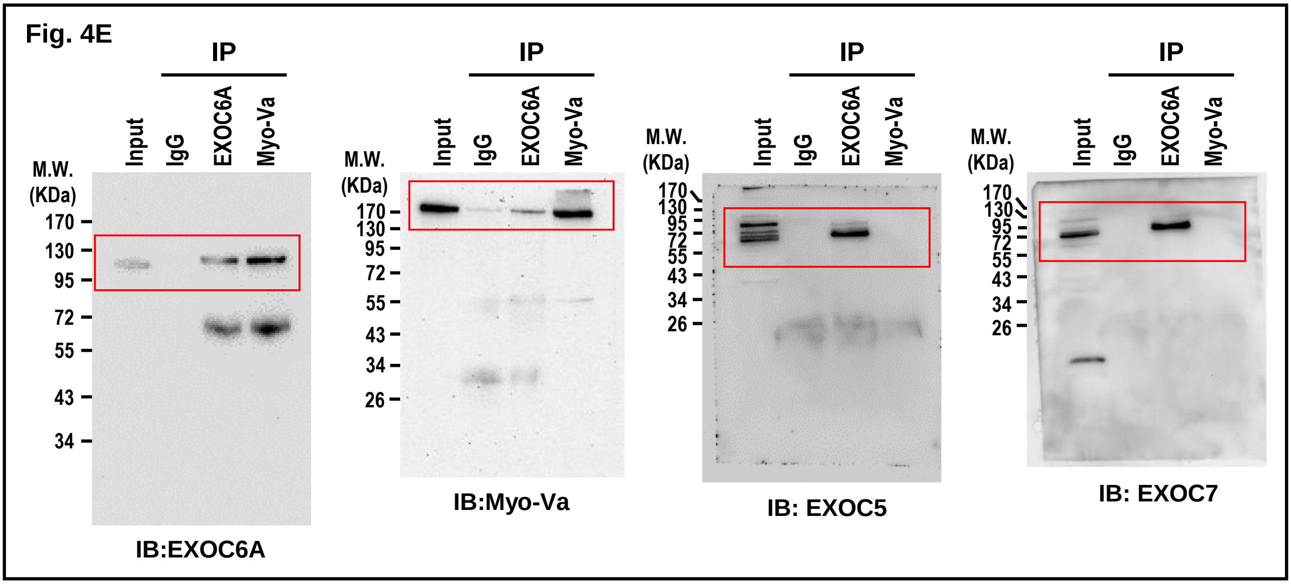

Fig. 4E
IP
Input
IgG
EXOC6A
Myo-Va
M.W.
(KDa)
170
130
95
72
55
43
34
IB:EXOC6A
IP
Input
IgG
EXOC6A
Myo-Va
M.W.
(KDa)
170
130
95
72
55
43
34
26
IB:Myo-Va
IP
Input
IgG
EXOC6A
Myo-Va
M.W.
(KDa)
170
130
95
72
55
43
34
26
IB: EXOC5
IP
Input
IgG
EXOC6A
Myo-Va
M.W.
(KDa)
170
130
95
72
55
43
34
26
IB: EXOC7

Source Data

Fig. 5B

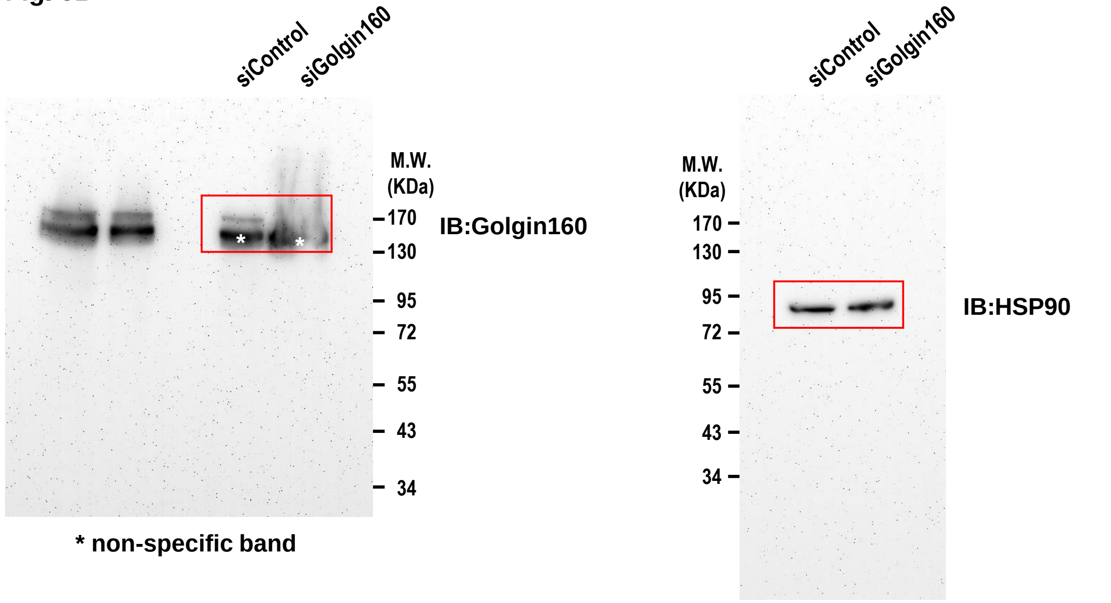

Fig. 5G

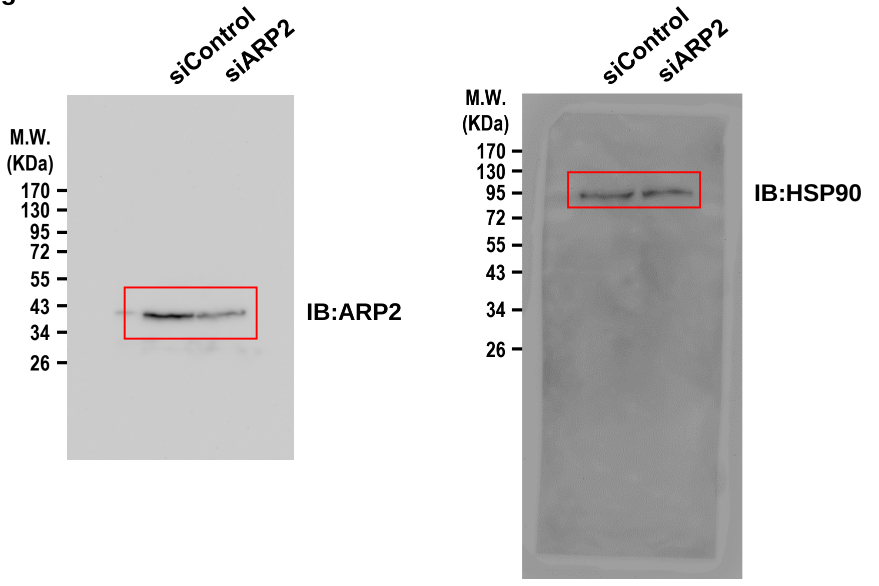

Supplement: 1 — Supplementary Figure 1. Correlation of spatial localization of EXOC6A and Myo-Va during the early stages of ciliogenesis. (A) RPE1-based mCherry-Myo-Va-GTD-inducible cells were treated with DOX for 24 h and serum starved for 0, 15, and 30 min. Fluorescence intensities of EXOC6A (green) or Myo-Va-GTD (red) signals within a 2 μm radius surrounding the Glu-tub-labeled centrioles (white) were quantified and are shown in B (EXOC6A) and C (Myo-Va-GTD). Correlation R (Pearson correlation coefficient) between EXOC6A and Myo-Va is shown in D. Correlation R was analyzed using Zeiss Zen blue software. Error bars in B–D represent mean ± s.d. from at least 3 independent experiments with 100 randomly selected cells. P-value was determined with one-way ANOVA. P < 0.05 was considered statistically significant. NS, not significant. Scale bars are 1 μm. Supplementary Figure 2. Live-cell imaging of GFP-EXOC6A co-localized with Myo-Va-GTD at the ciliary membrane (A) RPE1-based inducible cells expressing GFP-EXOC6A and mCherry-MyoVa-GTD were treated with Dox for 24 h, serum starved for 30 min, and SiR-tubulin was added to label centriole and axoneme. (B) Single channel image (green box in A) captured at the starting time point (0 sec). (C) Single channel image (red box in A) captured at frame 21 (197.577 sec). Scale bars are 1 μm. Supplementary Figure 3. EXOC6A deletion does not interfere with the removal of CP110 from the mother centriole. WT and EXOC6A KO cells were fixed 24 h after serum starvation and analyzed via fluorescence confocal microscopy using the indicated antibodies. (B) Percentages of cells with one or two CP110 dots are shown. Error bars represent the mean ± s.d. from at least 3 independent experiments with 100 cells per experiment. P-value was determined with two-tailed Student’s t-test. NS, not significant. Scale bars are 1 μm. Supplementary Figure 4. Low doses of Cytochalasin D (CytoD) promote cilia elongation, whereas higher concentrations (greater than 4 μM) inhibi [file NIHPP2025.07.25.666797V1-supplement-1.pdf]
